# Establishing consensus on ethics, traceability and biovigilance for medical products of human origin

**DOI:** 10.2471/BLT.21.285484

**Published:** 2021-10-28

**Authors:** Paul Ashford, Jeremy Chapman, Martin Hildebrandt, Luc Noël, Timothy Pruett, Ineke Slaper-Cortenbach, Diane Wilson

**Affiliations:** a International Council for Commonality in Blood Banking Automation, 1901 Orange Tree Lane Ste 200, Redlands, California 92374, United States of America (USA).; b Westmead Institute for Medical Research, University of Sydney, Sydney, Australia.; c University Hospital of Munich, Munich, Germany.; d Annecy-le-Vieux, France.; e Department of Surgery, University of Minnesota, Minneapolis, USA.; f Slaper-Cortenbach Biomedical Consultancy, de Bilt, Netherlands.; g Community Tissue Services, Dayton, USA.

Medical products of human origin are substances derived wholly or in part from donated human biological materials and intended for clinical application. They are obtained from a person, either deceased or living, and include blood, organs, cells, tissues, reproductive cells, breast milk and faecal microbiota. The unique relationship between the donor, processing events of such medical products and the recipient raises specific ethical issues in addition to biovigilance needs for traceability and surveillance by competent authorities.^1^

The spectrum of these products continues to grow. The use of pancreatic islets for glucose control is close to clinical application, and cell-based therapies for cancer and immunomodulation are expanding. Assessing and preserving the biologic qualities of medical products of human origin after donation is a challenge for their successful use. While many such donations result in a small number of products from a single donation (such as blood and reproductive cells), donation after death can yield thousands of products, ranging from organs and corneas with a time-limited value until implantation to decellularized bone chips with an almost indefinite shelf life. Many factors affect the quality of medical products of human origin, related to the donor, donation event and subsequent processing; thus, making all this information available at the point of clinical use would be advantageous. If the donor has an unrecognized transmissible disease (that is, not previously known to be transmissible through transplantation), a universal link is needed to facilitate prompt traceability of all the products derived from that donor to the product recipients. Conversely, when a recipient has an adverse event possibly caused by a medical product of human origin, it is critical that other products obtained from that donation event be quickly identified, other recipients identified and potentially harmful products quarantined, until risk is ascertained. Failure to quickly identify the recipients of the donated products and to quarantine nonutilized medical products of human origin results in unnecessary risk to the recipients.^2^ Additionally, the high demand for some of these products (most typically organs) has resulted in exploitive and criminal acquisition practices and inconsistent quality and safety standards. Clear linkage between donor and recipient is fundamental to all good practices of use of such products.

Traceability is essential to support the recall of medical products of human origin implicated in infectious transmission and to provide greater transparency of the chain of custody to help prevent the inappropriate procurement and use of such products.^3^ Standardization of terminology, coding and labelling is an important step in establishing reliable traceability systems. Significant progress has been made in achieving this objective through the widespread adoption of the ISBT 128 Standard, a standardized terminology, coding and labelling system for medical products of human origin that provides a system of globally unique identification together with a product coding system based on an internationally agreed terminology. According to the International Council for Commonality in Blood Banking Automation (ICCBBA), the international nongovernmental organization that manages ISBT 128,^4^ the system is currently used in more than 5700 facilities in 88 countries and its use continues to grow.

Three documents frame the ethical use of medical products of human origin. The World Health Organization (WHO) developed and endorsed the *Guiding principles on human organ transplantation* in 1991, and updated them to *Guiding principles on human cell, tissue and organ transplantation* in 2010.^5^ The Council of Europe *Convention on human rights and biomedicine*,^6^ which entered into force in December 1999, is the only international legally binding instrument on the protection of human rights in the biomedical field and includes a protocol concerning transplantation of organs and tissues of human origin. The convention addresses equitable access to transplantation services for patients, transparent rules for organ allocation, health and safety standards, the prohibition of financial gain by donors, and the need for donors, recipients, health professionals and the public to be properly informed of relevant medical, social, economic, ethical and legal implications. The scientific and professional community published the Declaration of Istanbul in 2008 to address concerns about organ trafficking, transplant tourism and transplant commercialism. As unethical activities around medical products of human origin continued, the Declaration was updated in 2018.^7^

Concerns have been raised about the clinical application of unproved cellular therapies that expose patients to unnecessary risks where there is no scientifically proven benefit, and the scientific and professional community is actively raising awareness of this issue.^8^ In January 2015, the document *Principles for global consensus on the donation and management of blood, blood components and medical products of human origin: report by the Secretariat* was sent to the WHO Executive Board. In response to this report, the Executive Board “requested that the Director-General convene consultations with Member States and international partners, to support the development of global consensus on guiding ethical principles for the donation and management of the mentioned medical products of human origin; good governance mechanisms; and common tools to ensure quality, safety and traceability, as well as equitable access and availability, as applicable.”^9^

This request was for the international community to develop the foundation for an integrated biovigilance system for medical products of human origin. Successful application of regulations, guidelines and principles depends on transparency, traceability, consistent regulatory oversight and the cooperation of the professional communities responsible for the procurement and clinical application of such products. Due to the global perspective of these products – with procurement, processing and clinical application potentially all occurring in different countries – international consensus on an ethical framework for medical products of human origin is needed.^10^ Such consensus, together with global standards for traceability, provides the basis for ensuring accountability.

The field of activity related to medical products of human origin is extremely broad, with different regulatory frameworks applying to these products within a single country or region, and a single product falling under different regulatory requirements in different countries. Additionally, professional communities typically restrict their concerns to specific categories of such products. The opportunities for broad collaboration are therefore limited.

The ICCBBA maintains technical advisory committees working closely with the relevant professional societies in each area of medical products of human origin, and with participation of regulators. As such, the council is uniquely positioned to support WHO in achieving its objectives with respect to these products by bringing together all the relevant parties to build an international consensus on ethical principles and traceability, and to develop the mechanisms to allow implementation of such a consensus to be monitored.

In 2019, the council organized an international forum with representation across all fields of medical products of human origin. The forum delegates identified 16 actions to sustain and improve on the current levels of traceability and ethical activity (Box 1) and the organizations best positioned to affect these recommendations were identified.^11^

Box 1 **Recommendations of the International Council for Commonality in Blood Banking Automation forum in 2019**

1. Governments should implement overarching principles, ethics and guidance across all medical products of human origin within specific jurisdictions.
2. WHO and regulatory committee/stakeholders should harmonize the legal oversight of all individual types of medical products of human origin across countries and/or continents.
3. Governments and professional organizations should increase donor safety, respect and privacy and where appropriate perform long-term follow-up.
4. The ICCBBA and professional organizations should improve traceability tools to ensure rapid tracking from a medical product of human origin to the donation event and ensure effectiveness through audit.
5. The ICCBBA, regulators and professional organizations should optimize the rapid tracing of all other medical products of human origin derived from a donation or donor for the purpose of inspection, quarantine or determination of recipient risk.
6. The ICCBBA, regulators, accreditation and standard-setting organizations, and professional organizations should encourage the adoption of globally unique donation identification and an international coding and labelling system for all medical products of human origin to enhance the traceability, quality and safety of medical products of human origin.
7. Governments and professional organizations should improve systems and skills to ensure recognition of potential adverse outcomes attributable to medical products of human origin and develop consistent reporting mechanisms that include patients and donor adverse events.
8. WHO, governments and professional organizations should establish a global system for adverse events reporting.
9. WHO, regulators and professional and patient organizations should improve biovigilance by enhancing adverse events reporting (also by patients) and systematic analysis of these events.
10. The ICCBBA, WHO/WHO Notify Library, regulators and professional organizations should build a global biovigilance system using new information technologies, while safeguarding privacy, for medical products of human origin starting from collection to clinical application.
11. WHO, regulators and professional organizations should improve dissemination of information on medical products of human origin-based therapies by rapid communication of reliable scientific results, encourage replication studies and the publication of clinical trials with negative results.
12. WHO, regulators and professional and patient organizations should define the safety and efficacy parameters for medical products of human origin-based therapies to accelerate the use in patients.
13. Regulators and professional organizations should assure the safety and efficacy of existing medical products of human origin-based therapies and evaluate clinical trials before acceptance of new or unproven medical products of human origin-based applications.
14. WHO, regulators and professional organizations should discuss and communicate the ethical challenges of new emerging technologies.
15. Governments, standard-setting and professional organizations should reach international consensus on quality, efficacy and ethical standards for medical products of human origin.
16. Governments, the ICCBBA, technology specialists and professional organizations should ensure that all traceability data related to a medical products of human origin-based therapy is incorporated into the electronic patient record in a consistent searchable manner.

ICCBBA: International Council for Commonality in Blood Banking Automation; WHO: World Health Organization.Source: Pruett T, et al.^11^

These recommendations were taken forward into relevant consultations including the European Commission Directorate-General for Health and Food Safety public consultation for the evaluation of the blood, tissues and cells legislation, and the United States Core Data for Interoperability submission system.

Since late 2019, the coronavirus disease 2019 (COVID-19) pandemic has further exacerbated the need for international standards that can respond to rapidly changing health care priorities. In response to the outbreak and rapid spread of COVID-19, blood transfusion organizations looked at the possibility of using convalescent plasma as an unproved treatment. The council was able to respond by assigning new ISBT 128 product description codes on an accelerated timescale. The use of these codes internationally made gathering of treatment data and subsequent analysis more efficient as products prepared in different countries were identified with the same product description codes and data could therefore be easily collated.

The topic of medical products of human origin safety remains under discussion, and a workshop organized in 2021 by the Pontifical Academy of Sciences and co-sponsored by WHO continued the debate on combating organ trafficking.^12^ This meeting recognized the need to establish registries and a system of traceability and vigilance to ensure the safety of donors and recipients as well as the efficacy and quality of organs.

Action to improve traceability tools is ongoing, including new standards for transmitting critical information using electronic messaging and developments to support the inclusion of transfusion and implant information into the electronic health record.

Progress in improving the safety of medical products of human origin depends on action; the council will work with WHO and the international community to develop tools to help countries and professional organizations assess the risks and expanding benefits of the use of such medical products.
